# Rubella vaccination in India: identifying broad consequences of vaccine introduction and key knowledge gaps

**DOI:** 10.1017/S0950268817002527

**Published:** 2017-12-04

**Authors:** A. K. WINTER, S. PRAMANIK, J. LESSLER, M. FERRARI, B. T. GRENFELL, C. J. E. METCALF

**Affiliations:** 1 Ecology and Evolutionary Biology, Princeton University, Princeton, NJ, USA; 2 Public Health Foundation of India, Gurgaon, Haryana, India; 3 Department of Epidemiology, Johns Hopkins Bloomberg School of Public Health, Baltimore, MD, USA; 4 IGDP in Ecology, The Pennsylvania State University, University Park, PA, USA

**Keywords:** Congenital rubella syndrome, CRS, India, rubella, vaccine

## Abstract

Rubella virus infection typically presents as a mild illness in children; however, infection during pregnancy may cause the birth of an infant with congenital rubella syndrome (CRS). As of February 2017, India began introducing rubella-containing vaccine (RCV) into the public-sector childhood vaccination programme. Low-level RCV coverage among children over several years can result in an increase in CRS incidence by increasing the average age of infection without sufficiently reducing rubella incidence. We evaluated the impact of RCV introduction on CRS incidence across India's heterogeneous demographic and epidemiological contexts. We used a deterministic age-structured model that reflects Indian states’ rural and urban area-specific demography and vaccination coverage levels to simulate rubella dynamics and estimate CRS incidence with and without RCV introduction to the public sector. Our analysis suggests that current low-level private-sector vaccination has already slightly increased the burden of CRS in India. We additionally found that the effect of public-sector RCV introduction depends on the basic reproductive number, *R*_0_, of rubella. If *R*_0_ is five, a value empirically estimated from an array of settings, CRS incidence post-RCV introduction will likely decrease. However, if *R*_0_ is seven or nine, some states may experience short-term or annual increases in CRS, even if a long-term total reduction in cases (30 years) is expected. Investment in population-based serological surveys and India's fever/rash surveillance system will be key to monitoring the success of the vaccination programme.

## INTRODUCTION

Rubella typically presents as a mild febrile rash illness in children. However, rubella infection in pregnant women can cause detrimental outcomes such as spontaneous abortion, fetal death and the birth of an infant with birth defects (i.e. congenital rubella syndrome (CRS)) [1]. Rubella-containing vaccine (RCV) is safe and effective [2]. High uptake can interrupt endemic rubella transmission and prevent CRS cases, as demonstrated in the World Health Organization (WHO) Americas region [3]. In contrast, countries in the WHO regions of Southeast Asia and Africa, which have been the slowest to add RCV to their national vaccination schedules, have the highest incidence of CRS, suffering 84% of the estimated 105 000 global incident CRS cases in 2010 [4]. India has the largest burden, with an estimated 40 000 cases [4].

Currently, RCV is only available in private-sector health facilities in India, where as few as 11% of children receive their immunisations [5]. As of February 2017, India began a phased introduction of RCV into its public-sector childhood Universal Immunization Programme (UIP). The introduction includes a one-time vaccination campaign targeting a wide age range of children followed by replacing the measles vaccine, administered within the current routine immunisation schedule, with a measles-rubella (MR) vaccine. High RCV uptake via public-sector vaccination has the potential to substantially reduce the global burden of CRS. However, if childhood RCV coverage falls below a critical threshold, CRS incidence can actually increase beyond rubella endemic CRS levels by increasing the average age of infection without sufficiently reducing rubella incidence [6–8]. A short-term (annual) increase in CRS post-RCV introduction, i.e. an increase in CRS in any given year, was observed in Greece after a rubella outbreak in 1993 [7] and in Costa Rica after a 1998–99 rubella outbreak [8, 9]; both these outbreaks followed a long period of low coverage. In theory, there is also a risk of long-term (30-year) increases in CRS [6, 10]; however, it has never been empirically observed.

The level of routine vaccination coverage required to reduce CRS depends dominantly on population birth rate and rubella transmissibility [11]. Previous mathematical models examining childhood immunisation programmes have suggested that 80% RCV coverage is sufficient to avoid long-term increases in CRS incidence post-RCV introduction across a range of demographic and epidemiological contexts [6, 11, 12]. No study to date has assessed the critical RCV coverage to avoid short-term increases in CRS, although it is likely to be at least 80%. Assuming that future routine RCV coverage rates will reflect current measles vaccine coverage rates in India, RCV coverage rates will likely range between 53% and 96% across Indian states’ rural and urban areas [5]; areas with coverage below 80% may be at risk of both long- and short-term increases in CRS [11].

The objectives of this strategic analysis were to evaluate the current burden of CRS in India while taking into account low-level private-sector RCV vaccination, and to explore the effect of introducing RCV into India's public-sector UIP on the short- and long-term incidence of CRS. To accomplish this, we simulated rubella dynamics across multiple vaccination scenarios (including no vaccination, private-sector vaccination and public-sector vaccination). We then compared CRS incidence over time between the different simulations. Given the diversity of demographic and potentially, epidemiological contexts across the subcontinent, we assessed these objectives sub-nationally by states’ rural and urban areas. This analysis additionally leveraged variation in demography to define threshold values of rubella epidemiological features (e.g. magnitude of rubella transmissibility) and RCV coverage necessary to avoid short- and long-term increases in CRS in the Indian context.

## METHODS

### Simulation of rubella dynamics

We simulated rubella transmission dynamics for rural and urban areas in 26 Indian states (52 areas, defined by the urban or rural portion of each state). We excluded three states: Goa and Sikkim because RCV has already been introduced into the public sector, and the newly formed state of Telangana. We used a deterministic age-structured mathematical model to simulate rubella transmission dynamics [10, 13–15]; its key feature is a matrix that at every time-step defines transitions from every possible epidemiological stage (maternally immune, susceptible, infected, recovered and vaccinated) and age combination, to every other possible epidemiological stage and age combination. The discrete time-step was set to about 2 weeks, the approximate generation time of rubella. For model details, see Supplementary Materials.

Data inputs required by the age-structured model include known features of rubella epidemiology, and states’ rural and urban area-specific demography. We assumed the basic reproductive number for rubella (i.e. *R*_0_, defined as the average number of people a typical infected individual will infect in a fully susceptible population) was five, as determined by a previous analysis of 40 African countries [11], and because it falls within the estimated range from India (3–9), based on serological data (Supplemental Fig. S2). Given that previous analyses have suggested that *R*_0_ affects CRS incidence [10], we conducted a sensitivity analysis for alternate values of *R*_0_ (7, 9 and 11) determined by an empirically estimated range [16–18]). We explored larger values of *R*_0_ to provide conservative predictions relative to the effects of vaccine introduction [11]. The model assumed assortative population age-mixing such that contact frequencies were proportional to those measured in the European POLYMOD study [19]. A sensitivity analysis was conducted using homogenous age-mixing. For details on model parameterisation, see Supplementary Materials.

### Vaccination scenarios

India's National Technical Advisory Group on Immunization asserts that RCV will be introduced in 2017 using two strategies: (i) a one-time MR vaccine catch-up campaign targeting individuals aged 9 months through 14 years old, and (ii) replacing the monovalent measles-containing vaccine (MCV) with the bivalent MR vaccine within the routine childhood vaccination schedule (i.e. administered to all children aged 9–12 and 16–24 months old). To explore the effect of RCV introduction, we simulated rubella dynamics across four vaccination scenarios, and compared CRS estimates. Two scenarios reflect the current vaccine use (no vaccination, low-level private-sector vaccination), one scenario reflects our best prediction of the planned RCV introduction as determined by the prevailing rates of MCV coverage, and one scenario is hypothetical to assess the WHO's minimum recommendation that all states maintain the critical rubella coverage threshold of 80% [12].

In scenario 1, the ‘no vaccine’ scenario, we simulated rubella disease dynamics over 56 years (simulation years 1991–2047, with the focal period for evaluation being the 30 years after 2017), assuming no vaccination in the public or private sector.

**Fig. 1.**
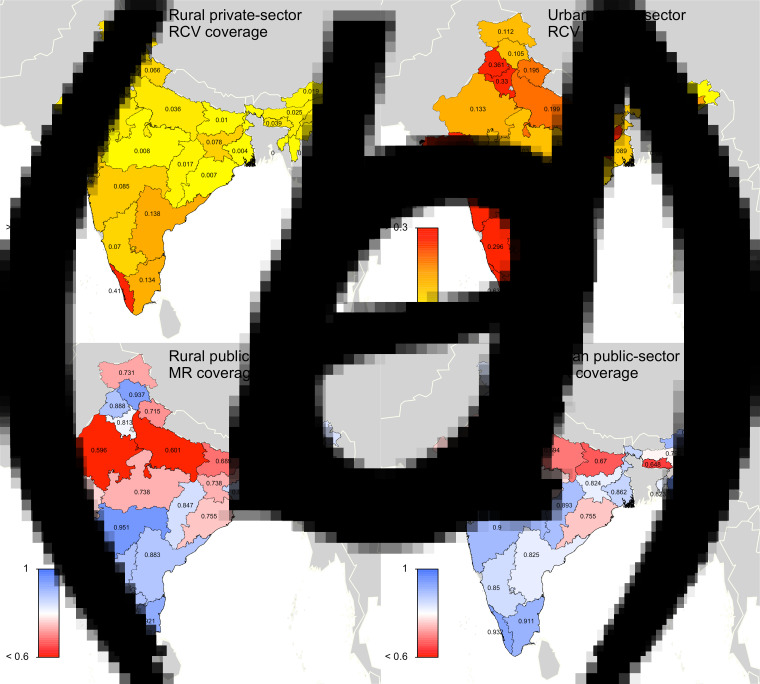
State-level covariates: (*a*) rural private-sector routine RCV coverage (as a proportion). (*b*) Urban private-sector routine RCV coverage (as a proportion). (*c*) Rural public-sector routine MR coverage (as a proportion). (*d*) Urban public-sector routine MR coverage (as a proportion). All coverage estimates were extracted from the Rapid Survey on Children 2013–14 [5]. To estimate private-sector routine RCV coverage, we assumed that any child who received their vaccinations in a private healthcare centre received RCV. To estimate public-sector routine MR coverage, we assumed that current MCV1 coverage estimates reflect future routine MR coverage estimates. See Supplemental Table S1 for a full list of coverage estimates for each simulated area.

In scenario 2, the ‘private-sector vaccine’ scenario, we simulated rubella disease dynamics as above, but allowed the private-sector routine RCV to be introduced in 1993 (when Serum Institute of India first launched India's measles-mumps-rubella (MMR) vaccine). We assumed that any child who received their vaccinations in a private healthcare centre received RCV, because the Indian Academy of Pediatrics recommends MMR vaccine for routine use at age 9 months, 15 months and 4–6 years old [20]. Area-specific estimates of the proportion of children who received vaccinations in private healthcare centres were extracted from the Rapid Survey on Children [5]. Private-sector coverage estimates were held constant between 1993 and 2047. Figure 1*a*, *b* displays the estimated private-sector RCV routine coverage for each area.

In scenario 3, the ‘60% catch-up + routine vaccine’ scenario, RCV was introduced in simulation year 2017, according to India's two-step implementation strategy. Scenario 3 assumed private-sector routine RCV coverage between 1993 and 2016 (private-sector estimates described above), assumed 60% coverage among 9-month through 14-year olds for the one-time MR catch-up campaign in 2017 (we also conducted a sensitivity analysis assuming 80% coverage, i.e. ‘80% catch-up + routine vaccine’ scenario), and held predicted routine MR coverage estimates constant for each state's rural and urban areas between 2017 and 2047. Given that the bivalent MR vaccine is replacing the monovalent MCV in India's public-sector childhood UIP, we assumed that MCV1 coverage estimates, extracted from the Rapid Survey on Children [5], will reflect future routine dose 1 MR coverage estimates for each states’ rural and urban areas. We were unable to characterise coverage of a second dose of MR given the lack of available data on MCV2, and therefore we did not include a second opportunity for MR vaccine in our model. Note that while this coverage estimate includes vaccines that take place in both the private and public sectors, we generally refer to this as the public-sector vaccination scenario to clarify that this scenario represents rubella and CRS estimates in the case that RCV is added to the public-sector vaccination schedule. Figure 1*c*, *d* displays the estimated public-sector MR routine coverage for each area.

In scenario 4, the ‘80% catch-up + 80% routine vaccine’ scenario, we assumed private-sector vaccination between 1993 and 2016, 80% coverage for the one-time MR catch-up campaign in 2017 and 80% routine dose 1 MR coverage estimates for all areas (even if predicted MR routine coverage was >80%) held constant between 2017 and 2047. This scenario is used to assess WHO's recommendation that all states maintain the critical rubella coverage threshold of 80% [12], which in part reflects the theoretical underpinning that the critical immunity threshold is 1 − (1/*R*_0_) [16] where *R*_0_ is five.

Each vaccination scenario was simulated independently for all 52 rural and urban areas. We estimated vaccine efficacy over age based on data extracted from [21], assuming a maximum efficacy of 97%.

### Evaluation of vaccination scenarios in the short- and long-term

Our model outputs the number of individuals in each age class and epidemiological stage at every time-step; therefore, we can estimate the predicted number of CRS cases for each time-step. The number of CRS incident cases for each time-step *t* was defined as
1
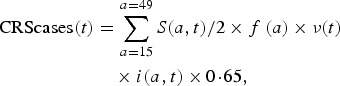

where *S*(*a*, *t*)/2 is the number of susceptible females at each age *a* and time-step *t* (we assumed exactly half of the susceptible population is female), and *f*(*a*) is the age-specific fertility rate per one woman. The *ν*(*t*) term is a correction ratio on *f*(*a*) to account for the fact that our simulations rely on crude birth rates to project births, rather than age-specific fertility rates; *ν*(*t*) is defined as the ratio of the total number of births estimated based on the crude birth rate at time-step *t* (i.e. *b*(*t*)) by the total number of births estimated from the age-specific fertility rate at time-step *t* (i.e. 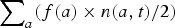, where *n*(*a*, *t*)/2 is the number of females in age class *a* and time-step *t*). The term *i*(*a*, *t*) is the probability of becoming infected with rubella over a 16-week period for each age *a* and time-step *t*. We assumed that the probability of CRS following rubella infection during the first 16 weeks of pregnancy was 0·65 [22].

We assessed the effect of RCV introduction on CRS incidence in the short-term by comparing summed CRS incident cases each year (every 24 time-steps) across vaccination scenarios, and in the long-term by comparing summed CRS incident cases over 30 years (720 time-steps between 2017 and 2047) across vaccination scenarios. We used a CRS incidence ratio as the measure of the effect, here defined for each specified length of time *l* (either annual or 30 years) as
2
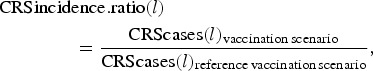

where CRScases(*l*) is the total number of CRS incident cases for a designated length of time.

All statistical analyses were conducted using R 3.2.4 [23].

### Determining *R*_0_, routine vaccination coverage and dynamical features indicative of a successful rubella vaccine introduction

We leveraged the diversity of demographic settings in India to identify threshold values for rubella *R*_0_ and routine RCV coverage that will result in a ‘successful’ RCV introduction. We also characterised dynamical features post-RCV introduction indicative of a ‘successful’ programme, focusing on honeymoon periods (i.e. periods of low incidence after a mass vaccination campaign resulting from the reduced size of the susceptible population, here, defined as the number of months between RCV introduction and a rubella outbreak of at least 5 cases per 100 000 population). We assessed three tiers of a ‘successful’ RCV introduction: (i) no long-term 30-year increase in CRS incidence, (ii) no short-term or annual increases in CRS incidence for 30 years and (iii) no rubella outbreak (defined as an annual rubella incidence of ⩽5 cases per 100 000). While *R*_0_ and RCV coverage estimates can be used prior to deployment as indicators of a ‘successful’ RCV introduction, the threshold value of the honeymoon period can only be used after a rubella outbreak has occurred (post-RCV introduction), as a warning signal for other areas of India. To identify threshold values for rubella *R*_0_ and routine RCV coverage, we determined the minimum values observed across all simulated areas in which the specified outcomes of a ‘successful’ introduction took place, and did not take place at any value greater. Threshold values for the length of the honeymoon period were associated with an assumed *R*_0_.

## RESULTS

**Fig. 2.**
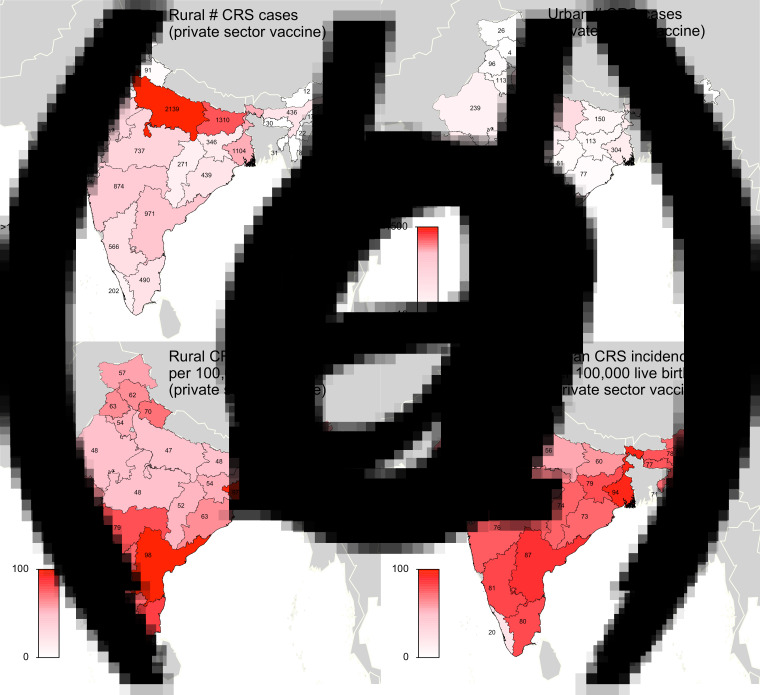
Results of simulated rubella dynamics assuming an *R*_0_ of 5 and private-sector vaccination since 1993: (*a*) Rural estimated 2016 number of CRS cases by state determined by ‘private-sector vaccine’ scenario. (*b*) Urban estimated 2016 number of CRS cases by state determined by ‘private-sector vaccine’ scenario. (*c*) Rural estimated 2016 CRS incidence per 100 000 live births by state determined by ‘private-sector vaccine’ scenario. (*d*) Urban estimated 2016 CRS incidence per 100 000 live births by state determined by ‘private-sector vaccine’ scenario. Broadly, rural areas experience higher burdens of CRS cases because they have larger populations, and urban areas have higher CRS incidence per 100 000 because they have higher private-sector coverage and lower birth rates. See Supplemental Table S2 for a full list of estimated CRS cases and incidence for each simulated area.

**Fig. 3.**
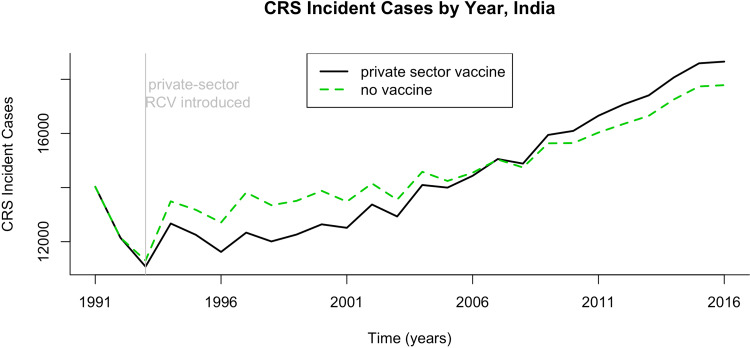
Results of simulated rubella dynamics assuming an *R*_0_ of 5. The number of CRS cases by year if private-sector vaccination is or is not taken into account, India 1991–2016.

We first evaluated the current burden of CRS in India while taking into account private-sector RCV vaccination. Our simulations suggested that if rubella *R*_0_ is five across India, and if private-sector vaccination was administered at coverage levels per [5], then approximately 19 000 children were born with CRS in India in 2016 (73 CRS cases per 100 000 live births). CRS incidence drops as the assumed rubella *R*_0_ increases; we estimated 11 000, 6700, 4300 incident CRS cases for *R*_0_ values of 7, 9 and 11, respectively. Figure 2 displays CRS incident cases and CRS incidence for each states’ rural and urban areas. We estimated that approximately 5·3% (1000/19 000) of CRS incident cases in 2016 can be attributed to private-sector vaccination (Fig. 3). Because the exact burden of CRS estimates depends on a large number of model assumptions (e.g. constant private-sector vaccination of RCV, assortative age-mixing [19], demographic assumptions discussed in Supplementary Materials and a 0·65 probability of CRS given rubella infection in first 16 weeks of pregnancy), we focused our analysis on a comparison between vaccination scenarios, rather than on the absolute CRS incidence values. Sensitivity analyses are displayed for assumptions that resulted in non-robust estimates in the vaccine comparative analysis (i.e. rubella *R*_0_ and age contact rates).

**Fig. 4.**
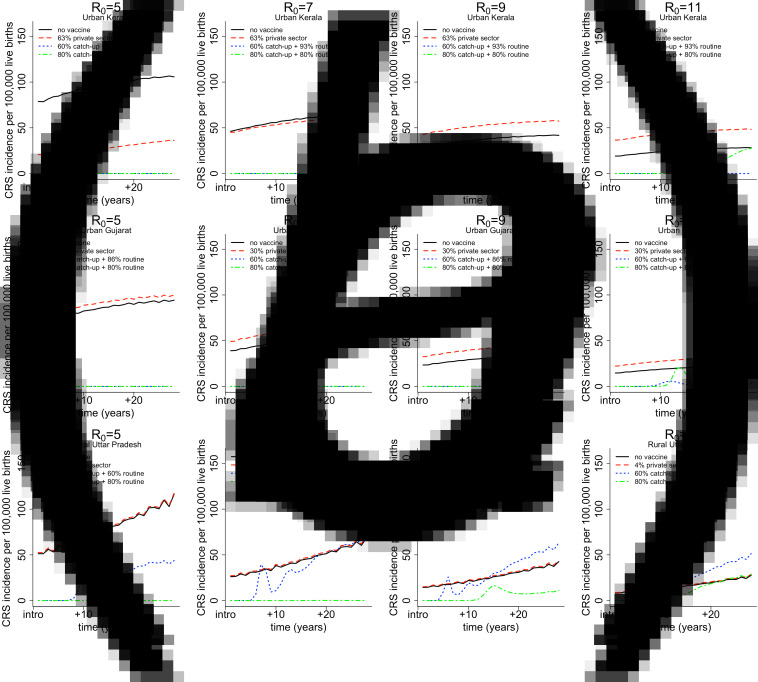
Results of simulated rubella dynamics by assumed *R*_0_ values across columns for (*a*) urban Kerala (high coverage, low birth rates) in row 1, (*b*) urban Gujarat (somewhat average coverage and birth rate) in row 2 and (*c*) rural Uttar Pradesh (low coverage, high birth rate) in row 3. The figures show CRS incidence per 100 000 live births over time for four vaccination scenarios by assumed *R*_0_ values across columns. The solid black lines represents the CRS incidence in the ‘no vaccine’ scenario. The dashed red line represents the CRS incidence in the ‘private-sector vaccine’ scenario; the estimated private-sector RCV coverage (as a proportion) is displayed in the legend for each area per [5]. The dotted blue line represents the CRS incidence in the ‘60% catch-up + routine vaccine’ scenario; the estimated public-sector routine MR coverage (as a proportion) is displayed in the legend for each area per [5]. The dashed and dotted green line represents the hypothetical ‘80% catch-up + 80% routine vaccine’ scenario, which is the critical RCV coverage threshold estimated per [12].

Figure 4 demonstrates simulation output of three heterogeneous areas (i.e. urban Kerala, urban Gujarat and rural Uttar Pradesh) showing the time-series of CRS incidence for the four vaccination scenarios and assumed values of *R*_0_. In addition to graphically illustrating the core outcomes, these figures reveal that CRS incidence is higher in the ‘private-sector vaccine’ scenario (represented by the red dashed line) compared with the ‘no vaccine’ scenario (represented by the black solid line) over time. This result holds true for all simulated areas beyond the three shown, with one exception: for relatively low *R*_0_ (5 or 7), urban Kerala does not experience an increase in the ‘private-sector vaccine’ scenario relative to the ‘no vaccine’ scenario. This occurs because urban Kerala has relatively high private-sector RCV coverage, and thus, if *R*_0_ is not high, this is sufficient to result in a decline in the CRS burden. In all other areas beyond urban Kerala, as a result of higher CRS incidence in the presence of private-sector coverage, reductions in CRS incidence post-RCV introduction into the public sector are larger in the presence of private-sector vaccination compared with no vaccination coverage.

Figure 4 also demonstrates the general finding for scenario 4, i.e. ‘80% catch-up + 80% routine vaccine’ scenario; the critical threshold of 80% (via catch-up and routine) is sufficient to prevent long-term increase in CRS incidence; and is sufficient to prevent any short-term or annual increases in CRS incidence given rubella's *R*_0_ is between five and nine. The same qualitative trend is observed in all other areas (results not shown).

We secondly explored the likely impact of public-sector RCV introduction on CRS incidence in the short- and long-term. Table 1 summarises the short-term effect of RCV introduction on CRS by displaying the number of years (out of 30) that the annual CRS incidence ratio was greater than one across assumed *R*_0_ values comparing ‘60% catch-up + routine vaccine’ scenario to ‘private-sector vaccine’ scenario. We found that as *R*_0_ decreases, the number of states and number of years with an annual CRS incidence ratio greater than one decreased (Table 1). At assumed *R*_0_ values of 11, 9, 7 and 5, we estimated that 24, 12, 4 and 0 areas experience short-term increases in CRS, respectively. See Supplemental Fig. S5 for the time-series of annual CRS incidence ratios by simulated areas across *R*_0_.

**Table 1.** Results of simulated rubella dynamics taking into account private-sector vaccination since 1993: the number of post-RCV introduction years (out of 30) in which the annual CRS incidence ratio of ‘60% catch-up + routine vaccine’ scenario compared with ‘private-sector vaccine’ scenario was greater than 1 by area and *R*_0_. As *R*_0_ increases, so does the number of simulated areas estimated to have an annual CRS incidence ratio greater than one in the ‘60% catch-up + routine vaccine’ scenario compared with the ‘private-sector vaccine’ scenario

States	R0:5	R0:7	R0:9	R0:11
Andhra Pradesh rural	0	0	0	0
Andhra Pradesh urban	0	0	0	0
Arunachal Pradesh rural	0	0	0	0
Arunachal Pradesh urban	0	0	0	0
Assam rural	0	0	0	14
Assam urban	0	0	0	5
Bihar rural	0	0	16	19
Bihar urban	0	0	14	16
Chhattisgarh rural	0	0	0	0
Chhattisgarh urban	0	0	0	0
Gujarat rural	0	0	0	13
Gujarat urban	0	0	0	0
Haryana rural	0	0	0	0
Haryana urban	0	0	0	9
Himachal Pradesh rural	0	0	0	0
Himachal Pradesh urban	0	0	0	0
Jammu & Kashmir rural	0	0	0	9
Jammu & Kashmir urban	0	0	0	0
Jharkhand rural	0	0	3	19
Jharkhand urban	0	0	0	0
Karnataka rural	0	0	0	0
Karnataka urban	0	0	0	0
Kerala rural	0	0	0	0
Kerala urban	0	0	0	0
Madhya Pradesh rural	0	0	13	17
Madhya Pradesh urban	0	0	0	11
Maharashtra rural	0	0	0	0
Maharashtra urban	0	0	0	0
Manipur rural	0	0	0	12
Manipur urban	0	0	0	10
Meghalaya rural	0	0	14	18
Meghalaya urban	0	0	8	14
Mizoram rural	0	0	0	0
Mizoram urban	0	0	0	0
Nagaland rural	0	12	16	18
Nagaland urban	0	1	12	17
Odisha rural	0	0	0	14
Odisha urban	0	0	0	10
Punjab rural	0	0	0	0
Punjab urban	0	0	0	0
Rajasthan rural	0	14	19	19
Rajasthan urban	0	0	0	13
Tamil Nadu rural	0	0	0	0
Tamil Nadu urban	0	0	0	0
Tripura rural	0	0	0	12
Tripura urban	0	0	0	0
Uttar Pradesh rural	0	8	17	18
Uttar Pradesh urban	0	0	8	15
Uttarakhand rural	0	0	3	15
Uttarakhand urban	0	0	0	0
West Bengal rural	0	0	0	0
West Bengal urban	0	0	0	0

**Fig. 5.**
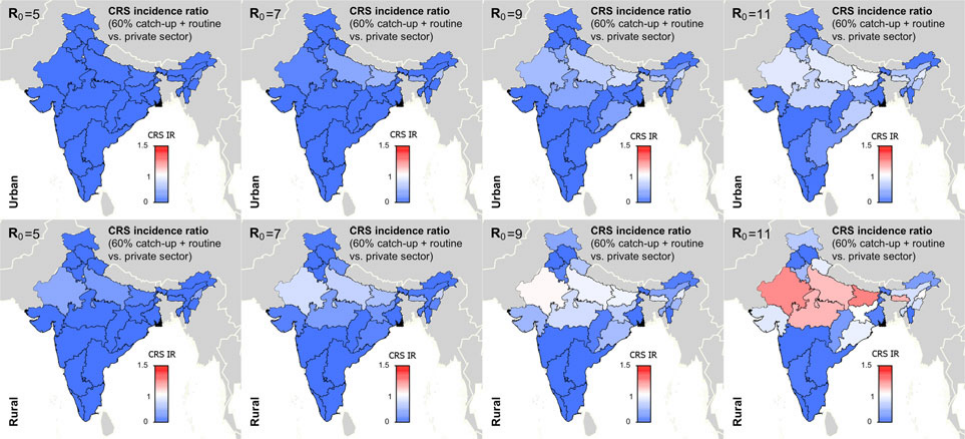
Results of simulated rubella dynamics taking into account private-sector vaccination since 1993: 30-year CRS incidence ratio (IR) of ‘60% catch-up + routine vaccine’ scenario compared with ‘private-sector vaccine’ scenario across all states by rural and urban areas and *R*_0_. Shades of blue represent a CRS incidence ratio less than one, the colour white represents a CRS incidence ratio of one, and shades of red represent a CRS incidence ratio of greater than one. At *R*_0_ = 11, we estimated six areas may experience a long-term increase in CRS post-RCV introduction (i.e. rural areas in Rajasthan, Uttar Pradesh, Madhya Pradesh, Bihar, Meghalaya and Nagaland); and at *R*_0_ = 9, rural Rajasthan was estimated to have a long-term increase in CRS. As *R*_0_ increases, so does the number of simulated areas estimated to have a long-term CRS incidence ratio greater than one in the ‘60% catch-up + routine vaccine’ scenario compared with the ‘private-sector vaccine’ scenario.

Figure 5 summarises the long-term effect of RCV introduction on CRS across *R*_0_. We found that if *R*_0_ is five or seven, then current vaccination coverage levels are sufficient to prevent long-term increases in CRS (Fig. 5). If *R*_0_ is nine, only rural Rajasthan was estimated to have an increase in CRS over 30-year post-RCV introduction, and if *R*_0_ is as high as 11, six areas were estimated to have a long-term increase in CRS post-RCV introduction (Fig. 5). The results of scenario 3 sensitivity analysis shows that the effect of RCV introduction on CRS incidence in the short- and long-term were robust to the assumed catch-up campaign coverage (60% *vs.* 80%) given estimated routine coverage rates per simulated area (Supplemental Figs S6 and S7).

Figures 4 and 5 and Table 1 show that RCV introduction is most effective at reducing CRS burden in the short- and long-term at lower values of *R*_0_. This is because (i) the burden of CRS is higher for lower values of *R*_0_, thereby increasing the opportunity for large reductions in CRS post-RCV introduction, and (ii) the critical RCV coverage threshold decreases as *R*_0_ decreases, thereby increasing the probability of a reduction in cases across states’ rural and urban areas given current vaccination coverage rates [16].

The sensitivity analysis of age-contact rates showed that compared with assortative age-mixing determined by the POLYMOD study [19], homogenous age-mixing resulted in higher current estimates of CRS (Supplemental Fig. S8), such that public-sector RCV introduction resulted in larger declines in CRS incidence (Fig. 5 *vs.* Supplemental Fig. S9). Therefore, our primary results, which assumed age assortative mixing, reflect a conservative assessment of the benefit of vaccination in reducing CRS incidence in the short- and long-term.

Table 2 displays the threshold values for the *R*_0_ of rubella, routine RCV coverage and the honeymoon period length for each of the three outcomes defined above as a ‘successful’ RCV introduction, and the data that can be used to anticipate them. As demonstrated in Fig. 5 and Table 1, the *R*_0_ threshold values for no increase in CRS incidence in the long- and short-term are five and seven, respectively. We additionally found that the minimum routine RCV coverage assuming *R*_0_ is 11 must be at least 76% and 82% in order to avoid a long-term or any short-term increases in CRS, respectively; however, the critical vaccination coverage will be lower as *R*_0_ decreases (Table 2).

**Table 2.** Threshold values suggested by our analysis and sources of data that can be used to evaluate three tiers of a ‘successful’ RCV introduction into the public sector in India: a long-term 30-year CRS incidence ratio (IR) <1, all short-term or annual CRS incidence ratio (IR) <1 and all annual rubella incidence <5 cases per 100 000 live births (the results are determined by the ‘60% catch-up + routine vaccine’ scenario)

Outcomes	Threshold values	Data sources
30-year CRS IR <1	5 ⩽ *R*_0_ ⩽ 7	• Age-stratified serological surveys [24–26] • Rubella age-incidence surveillance data [16, 24, 25, 27]
	Routine RCV vaccine coverage ⩾0·76 (if *R*_0_ = 11); ⩾0·60 (if *R*_0_ = 9)	• Pre- and post-vaccination serological surveys [28] • Post-vaccination serological testing that can distinguish vaccination and natural immunity • Vaccine coverage survey data [5] • Vaccine administration data [29]
	Average number of months until rubella outbreak post-vaccine introduction ⩾107 months	• Rubella incidence surveillance data
All annual CRS IR <1	*R*_0_ = 5	• Age-stratified serological surveys [24–26] • Rubella age-incidence surveillance data [16, 24, 25, 27]
	Routine RCV vaccine coverage ⩾0·82 (if *R*_0_ = 11); ⩾0·77 (if *R*_0_ = 9); ⩾0·70 (if *R*_0_ = 7)	• Pre- and post-vaccination serological surveys [28] • Post-vaccination serological testing that can distinguish vaccination and natural immunity • Vaccine coverage survey data [5] • Vaccine administration data [29]
	Average number of months until rubella outbreak post-vaccine introduction ⩾127 months	• Rubella incidence surveillance data
All annual rubella incidence <5	Routine RCV vaccine coverage ⩾0·95 (if *R*_0_ = 11); ⩾0·89 (if *R*_0_ = 9); ⩾0·85 (if *R*_0_ = 7); ⩾0·76 (if *R*_0_ = 5)	• Pre- and post-vaccination serological surveys [28] • Post-vaccination serological testing that can distinguish vaccination and natural immunity • Vaccine coverage survey data [5] • Vaccine administration data [29]

Table 2 also displays the threshold values for the honeymoon period length. We estimated that an average honeymoon period of 107 and 127 months represent the threshold honeymoon periods associated with long-term CRS incidence ratio less than one and all short-term CRS incidence ratios less than one, respectively. See Supplemental Table S3 for the estimated length of the honeymoon period for each states’ rural and urban areas by assumed *R*_0_ value. Given that the length of the honeymoon period varies across sub-populations, early rubella outbreaks can provide predictive power for other sub-population outbreaks. While there is currently no reliable national surveillance and registry in India [30, 31], improved surveillance is an aim within India's National Operational Guidelines for the introduction of RCV [32]. We found that fever/rash surveillance would need to be strengthened, such that at least 27% of all rubella cases actually occurring are reported in order to capture the average post-honeymoon period rubella outbreak across a range of *R*_0_ values (Supplemental Table S3). The direct policy implications, however, of this finding for India's measles-rash surveillance system is an area for future research and will depend on the number and location of reporting sites within each state, in addition to knowledge of spatial connectivity across the country.

## DISCUSSION

There is increasing recognition of the importance of moving beyond country-scale analyses and considering heterogeneity within countries to evaluate public health interventions [33, 34]. In this analysis, we provide a detailed state by rural and urban area model-based strategic analysis of the current burden of CRS, taking into account private-sector coverage, and the impact of RCV introduction into India's public-sector childhood vaccination programme.

Our analysis confirmed [22] that low-level RCV coverage, represented by private-sector RCV routine coverage levels in India (<63% excluding urban Kerala [5]), results in increases in CRS incidence by approximately 5·5%, relative to no vaccination. This finding reinforces the importance of introducing RCV into the public sector to drive down potential increases in CRS from private-sector vaccination.

Our analysis additionally confirmed previous theoretical findings of the potential for long-term increases in CRS given insufficient RCV coverage [6]. However, this result emerged only if the *R*_0_ of rubella across India is 11 (with the one exception of rural Rajasthan when *R*_0_ = 9). Given the lack of observed evidence of long-term increases in CRS globally, the wealth of empirical data analysis that estimate rubella *R*_0_ <11 [11, 16, 18], and our estimates of rubella *R*_0_ across India ranging between 3 and 9 (Supplemental Fig. S2), our results suggest that long-term increases in CRS are unlikely in India post-RCV introduction. However, short-term or annual increases in CRS, due to post-honeymoon period outbreaks of rubella, remain an issue of concern.

The effect of RCV introduction into India's public-sector UIP on short-term increases in CRS will depend on the magnitude of rubella transmission across India. If we assume that the *R*_0_ of rubella across India is five (the median estimate across the continent of Africa [11]), we find that RCV introduction will result in annual decreases in the incidence of CRS across all states. However, if the *R*_0_ of rubella is seven (as estimated in some European countries [16, 18]), then RCV introduction may result in short-term increases in CRS incidence in areas where predicted routine RCV coverage is <70% (i.e. rural Rajasthan, rural Uttar Pradesh and Nagaland). If the *R*_0_ of rubella is nine, our results suggest that eight states (12 areas), where predicted routine RCV coverage is <82%, may experience annual increases in CRS. Although we did estimate the *R*_0_ of rubella to be as high as nine in Vellore, Tamil Nadu in 1999 (Supplemental Fig. S2), it is unknown to what degree private-sector vaccination may be biasing this estimate upwards.

We confirmed that the current WHO recommended critical RCV threshold of 80% routine coverage [12] is sufficient to prevent a long-term increase in CRS incidence for all simulated *R*_0_ values, and prevent any short-term or annual increases in CRS if rubella's *R*_0_ is between 5 and 9, across India's heterogeneous demographic and epidemiological contexts.

Given the limited generalisability and spatial representation of serological surveys to date in India [4, 30], and the lack of a reliable national surveillance system for rubella in India [30, 31], it is difficult to evaluate the true *R*_0_ for rubella and its distribution across India. As such, population-based rubella serological surveys or non-age biased incidence data are needed to inform unknown transmission drivers such as *R*_0_ [24, 25]. Additionally, these types of data sources will be invaluable in the future to evaluate the success of the RCV introduction, and to determine the need for future supplemental immunisation activities (SIAs).

We did not model the opportunity for a second dose of the MR vaccine given the lack of available data; thus, we assumed the probability of the second dose of the vaccine to immunise children with vaccine failure from dose 1 and the probability of the second dose of vaccine to be administered to children missed by the first dose of vaccine was zero. This conservative approach results in an underestimation of RCV routine coverage estimates. However, qualitatively our results are unlikely to change much assuming high correlation between receiving the first and second doses of the vaccine. We predicted that RCV public-sector routine coverage estimates are equal to current MCV routine coverage estimates, and will remain constant over time. Given India's dedication to eliminating measles [35], our MR routine coverage estimates likely underestimate future estimates. Our results, therefore, are conservative and may overestimate the number of CRS cases post-RCV introduction and underestimate the benefits of RCV introduction. We additionally did not take into account the potential for periodic SIAs to be conducted following the initial catch-up campaign. These campaigns have been shown to successfully reduce susceptible populations [3], and would likely lengthen the honeymoon periods and potentially even prevent the return of endemic rubella dependent upon the degree of increases in routine immunisation and the coverage and age target of the periodic SIAs.

While our deterministic model enabled us to explore RCV introduction at the sub-national level and highlight threshold values for a successful introduction, our model did not evaluate additional complex dynamical features of rubella that can shape transmission dynamics and CRS incidence [36, 37]. For example, we did not account for extinction and recolonisation dynamics suggested to explain rubella transmission patterns in Peru [36]. As a result, a subset of remote Indian communities may experience an increase in CRS incidence post-RCV introduction, even if estimated thresholds for *R*_0_ and RCV coverage are met. Detailed spatial connectivity data and spatially resolved rubella incidence data are necessary to assess these metapopulation dynamics. Metapopulation dynamics of rubella are an interesting direction for future work in India, specifically in light of the phased spatial roll-out of RCV that is currently taking place [32]. We also did not evaluate the impact of the timing of RCV introduction on rubella short-term transient dynamics and the honeymoon period duration [37]. Because the *R*_0_ of rubella likely falls below the threshold value for complex multi-annual cycles [38], our results are likely to be broadly robust to this aspect, but it remains an interesting direction for further investigation.

The reliability of model-based simulations depends on model structure, model assumptions and parameter estimates. We assumed seasonal forcing of rubella infections following a sinusoidal pattern determined by a range of settings [10, 36, 39]; despite a lack of direct evidence from India, the degree of forcing has a negligible effect on CRS estimates [10]. We assumed that the rate of contact by age for India was proportional to the POLYMOD study [19]. Analyses in other countries qualitatively suggest similar age-assortative patterns [19, 40, 41]; however, data from our focal populations would strengthen the analysis. We assume that measures of age contacts are directly related to measures of transmission; however, despite best efforts, these have only been circumstantially linked [42, 43]. The question of whether alternate patterns of contact might further improve model performance is largely intractable given issues of identifiability, and this remains an active area of research. Our age-mixing sensitivity analysis suggests that our broader conclusions of the effect of RCV introduction are conservative compared with homogenous age-mixing. Estimated private- and public-sector RCV coverage estimates extracted from [5] are prone to bias from mother's recall, the accuracy of vaccination cards and the rate at which children who receive vaccination in the private sector did not receive RCV. Despite these assumptions and caveats, disease dynamic projections are important analytical tools for policy makers, particularly in data-poor contexts.

Our strategic analysis across an array of vaccination scenarios provides general optimism that RCV introduction into India's public-sector UIP will result in decreases in CRS incidence, specifically in light of low-level private-sector RCV coverage. However, the diversity of outcomes suggested by our results, which stem largely from uncertainty linked to rubella transmission in India, indicates that India's readiness and flexibility to administer SIAs and capacity to increase routine vaccination coverage rates (particularly in rural areas in northern India) will be critical. Moving forward, age-structured serological surveys and a strengthened fever/rash surveillance system will be key for the evaluation of the MR vaccination campaigns and the potential need for SIAs to not only control the spread of rubella, but also measles.

## Supplementary material

For supplementary material accompanying this paper visit https://doi.org/10.1017/S0950268817002527. click here to view supplementary material
