# Supplementary material for: Rubella vaccination in India: identifying broad consequences of vaccine introduction and key knowledge gaps
Source: Epidemiol Infect. 2017 Dec 4;146(1):65–77. doi: 10.1017/S0950268817002527 (PMC6024169; doi:10.1017/S0950268817002527)
Supplement: Supplementary file 1 [file S0950268817002527sup001.zip › S0950268817002527sup001/v26.Supplement.docx]

Supplementary Material

*Epidemiology and Infection*

“Rubella Vaccination in India: Identifying broad consequences of vaccine introduction and key knowledge gaps”

A.K. Winter, S. Pramanik, J. Lessler, M. Ferrari, B.T. Grenfell, and

C.J.E. Metcalf

# Supplementary Methods

- 1. *Model Structure (originally presented by Metcalf et al. (2012) [1])*

To simulate rubella transmission dynamics for rural and urban areas in 26 Indian states (52 total areas),we used a discrete-time model that incorporates both epidemic and demographic transitions, building on theory introduced in [2, 3]. The model structure described here is as originally presented in [1], and used to described rubella dynamics in [4]. We structured the population into 5 epidemiological stages (maternally immune ‘M’, susceptible ‘S’, infected ‘I’, recovered ‘R’, and vaccinated ‘V’, taken to indicate the effectively vaccinated), and 320 age classes (monthly strata up to age 20 years, and yearly strata until age 100 years). The key feature of the model is a large matrix that at every time-step defines transitions from every possible epidemiological stage and age class combination, to every other possible epidemiological stage and age class combination.

We take a two step approach to describe the large transition matrix. Step one is to define epidemiological transitions, ignoring demographic transitions (aging and survival). Matrix **A***a,t*, captures transitions between each epidemiological stage within each age class *a* and time-step *t*, where the time-step *t* was set to the approximate generation time of rubella at about 2 weeks (i.e., there are 24 infection generations per year). Matrix **A***a,t* is defined as,


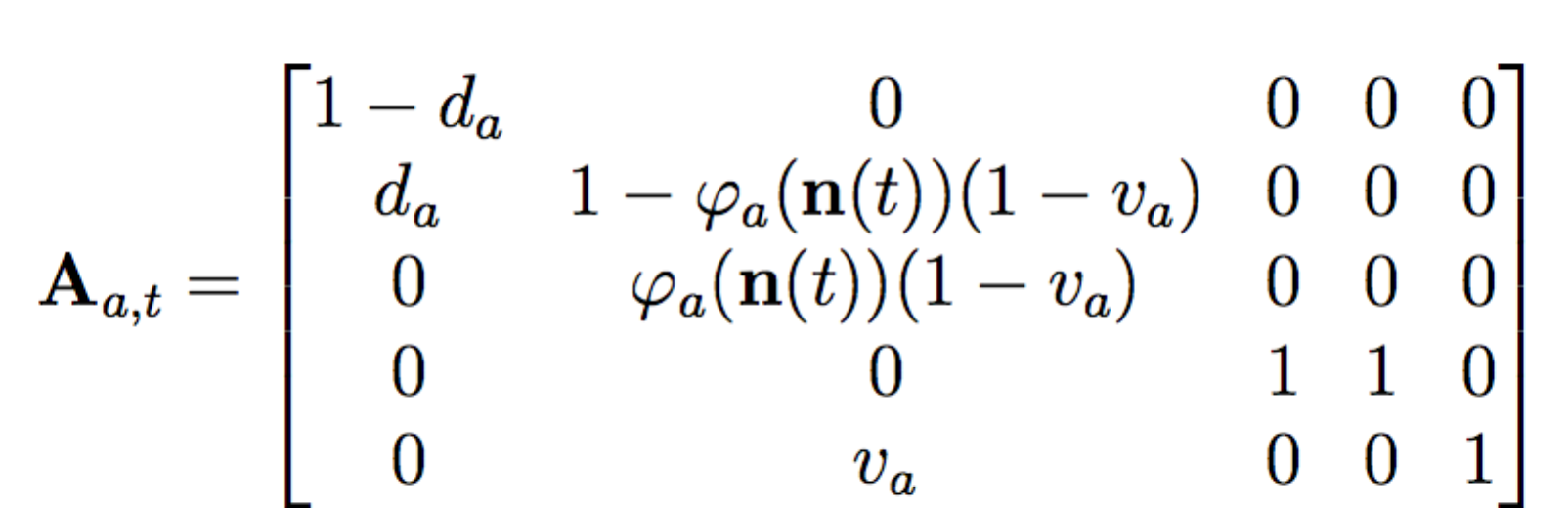


The five rows from left to right and columns from top to bottom represent the ‘M’, ‘S’, ‘I’, ‘R’, and ‘V’ epidemiological stages, respectively. In the transition

matrix, *da* is the probability of losing maternal immunity by age class *a*, *ϕa* is the probability an individual in age class *a* becomes infected, and *va* is the probability an individual in age class *a* is successfully vaccinated. The probability of infection, Φ (also called the force of infection, FOI) is a function of **n**(*t*), a vector describing the population at time *t*, defined as,

according to

$$\phi_{a}\left( \boldsymbol{n}\left( t \right) \right)= 1-exp(\frac{-\sum_{j} \beta_{a,j,t}I_{j,t}^{\gamma}}{\sum\boldsymbol{n}\left( t \right)})$$

**n**(*t*) = (*M*1*,t, S*1*,t, I*1*,t, R*1*,t, V*1*,t, M*2*,t, ...Vz,t*)*T* (1)

(2)

where *z* is the total number of age classes (here *z*=320), *βa,j,t* is the rate of transmission between individuals in age class *a* and *j* at time-step *t*, as known as the Who-Aquires-Infection-From-Whom (WAIFW) matrix, and $I_{j,t}^{\gamma}$ is the number of infected individuals in age class *j* and time-step *t*, while *γ* captures the non-modeled heterogeneities in age mixing [5, 6] and the effects of discretization of the underlying continuous process [7]. In this analysis, we fixed *γ* at 0.97 [5], because discrete-time models that do not incorporate this exponent result in unrealistically unstable dynamics prone to frequent extinction. Given that rubella transmission is frequency dependent, we divided the number of infected individuals in each age class by the total population size [8].

The second step is to construct the full transition matrix, **A**(**n**(*t*)), that includes both epidemiological transitions captured in matrix **A***a,t*, and demographic transitions (aging and survival). This matrix is used to project the entire population forwards via aging, mortality, and infection dynamics and is defined as,


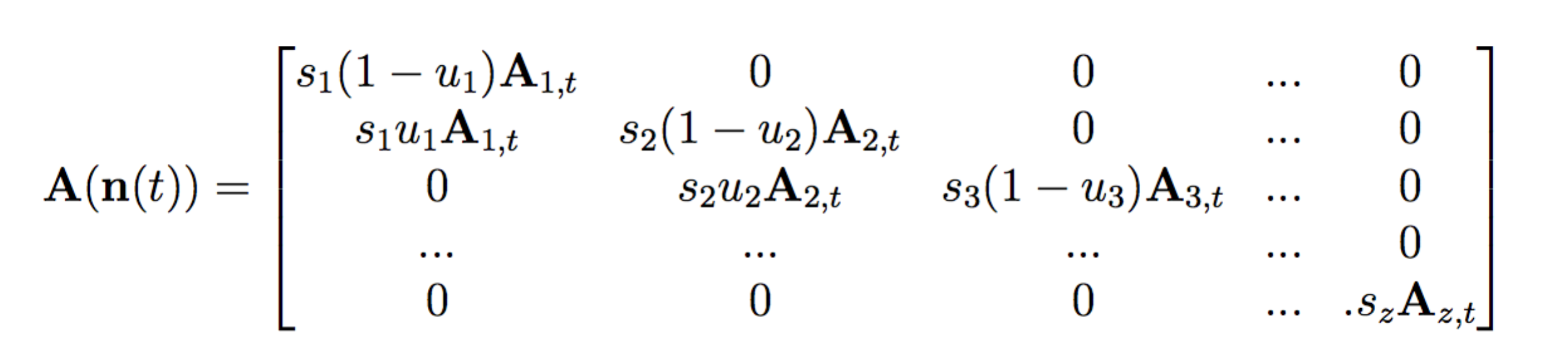


where *sa* is the probability that an individual in age class *a* survives to the next time step, *ua* is the rate of aging out of age class *a*, and **A**1*,t*,**A**2*,t*, etc.,

are the defined in equation 1. We assumed constant rate of aging into the next age class (i.e., *ua* = 1 / (length of age class *a* in years times 24)). The dynamics of the whole population can then be projected forward in time, such that

**n**(*t* + 1) = **A**(**n**(*t*))**n**(*t*) + **B***t* (3)

where **B***t* is a vector representing the number of births at time *t*, defined as,

**B***t* = (*bt,* 0*,* 0*, ...*0)*T .* (4)

- 1. *Parameterizing the Model*

Given the absence of detailed data on rubella dynamics in India, we combined estimates of local demographic rates with broadly known rubella epidemiology in order to estimate features of rubella dynamics in India. The following sections describe our parameterization of epidemiological and demographic features of this model.

- - 1. *Epidemiological Parameters*

The duration of protection by rubella maternal antibodies ranges between 3 and 9 months [9, 10]; accordingly, we modeled the probability of remaining in the maternally immune epidemiological stage over age (1-*da*) as an exponential decay function with a constant rate of 0.95 per month (Supplemental Figure S1a).

*−*

We used published rubella serological data in India to estimate a potential range for the basic reproductive number (i.e., *R*0, defined as the average number of people a ‘typical’ infected individual will infect in a fully susceptible population) of rubella in India. Age-structured serological surveys of rubella Immunoglobulin G (IgG) antibodies are a tool for the direct characterization of the age immunity profile. We limited our analysis of published rubella serological surveys in India to works cited in a 2016 review of serological data [11]. Published serological survey data that had three or fewer age groups were excluded from this analysis, resulting is 6 datasets [12, 13, 14, 15, 16, 11]. In each dataset, we estimated the average age of infection by i) fitting a cubic smoothing spline to the proportion seropositive by age, ii) estimating the derivative of the fitted curve (scaled to one) to obtain an estimate of the distribution of rubella cases by age (assuming no RCV vaccination), and iii) calculating the numerical mean of the probability

density curve. *R*0 can then be defined for each sample population as 1 +*L/A*, where *L* is the life expectancy at birth, and *A* is the average age or infection [17]. Estimates for life expectancy at birth were extracted from the World Bank [18] for the country of India and matched to the year each serological dataset was collected. We estimated that the *R*0 of rubella ranges between 3 and 9 (Supplemental Figure S2). While this analysis gives us a sense of the *R*0 of rubella in India, the data has limitations related to small scope of areas in India represented and the generalizability of the samples to the country. Additionally, the analysis has limitation because the simple calculation of *R*0 assumes an untrue stable population, and constant force of infection across age [17, 19]. The calculation of *R*0 also assumes no rubella vaccination coverage, which for the five out of six studies that take place before 1993 (when Serum Institute of India first launched India’s Measles-Mumps-Rubella vaccine), this is a good assumption [12, 13, 14, 15, 16]. However, the dataset from Vellore, Tamil Nadu was collected in 1999 [11], therefore not taking into account potential private sector vaccination will result in an over-estimate of *R*0, although magnitude of bias is unknown.

Estimates of *R*0 for rubella based on population or community-based serological data have ranged from 3 in European countries [19] to 11.8 in

Addis Ababa, Ethiopia [20]. An in-depth empirical analysis of rubella incidence data from 40 African countries estimated that the median *R*0 of rubella across the continent of Africa was 5.2 (90% CI 4.0-6.7) [21]. Taking into ac- count the range of *R*0 estimated for India above, and the robust empirical finding across heterogeneous African settings [21], we assumed an *R*0 of 5 for our analysis. Given that *R*0 can vary between settings and is known to affect CRS outcomes [4], we additionally ran a sensitivity analysis for other values of *R*0 (i.e., 7, 9, and 11). We explored larger values of *R*0 in order to provide conservative predictions relative to the effects of vaccine introduction [21], and went up to 11 as an upper bound based on the finding in Addis Ababa [20].

The model assumed seasonal forcing [22, 4, 8, 23, 24], where intensity of transmission varied over each year (affecting all age groups the same). Transmission to individuals in age class *a*, from individuals in age class *j* for each time-step *t* are defined by *βa,j,t* = *β*¯*a,j* (1 + *α* cos(2*πt*)), where *β*¯*a,j* is mean transmission from individuals in age class *j* to age class *a*, and *α* is a parameter controlling the magnitude of seasonal fluctuations. Empirical estimates from large-scale qualitative studies generally converge on assortative age-contact patterns relevant for infections transmitted via the respiratory

route, specifically among school ages [25, 26, 27]. Accordingly, we assumed age-specific contact patterns proportional to those characterized in the European POLYMOD study based on diary entries [25]. We calculated the mean WAIFW matrix (i.e., *β*¯*a,j* ) by rescaling the Great Britain age contact rates observed in the POLYMOD study to reflect the assumed *R*0 of rubella (defined in the previous paragraph). Given the importance of age contact patterns on transmission [19], a sensitivity analysis was conducted using homogenous age transmission rates (again rescaled to reflect the assumed *R*0 value) such that individuals are no more likely to be infected by a member of a different age group than their own. Previous validation of this model has shown that model results were robust to the magnitude of seasonal fluctuations [4]; we set *α* to 0.15 and held it constant over time (Supplemental Figure S1b).

The probability of effective vaccination for individuals in age class *a* (i.e., *va*) was obtained by multiplying the probability of access to vaccination by age (probability of access to routine vaccination is based on estimates from Zambia [28]; probability of access to SIA vaccination was assumed to be 1 for all ages in the target age range), by a vaccination efficacy rate over age (based on data extracted from [29]). The probability of access to routine vaccination over age was scaled to reflect our assumed coverage. And, we forced vaccine efficacy over age to saturate at 97%.

Rubella epidemiological parameter inputs were not area-specific and were assumed across all states’ rural and urban areas.

- - 1. *Demographic Parameters*

To simulate rubella dynamics across each states’ rural and urban areas, we first needed area-specific rubella endemic populations (i.e., **n**(1)). We began with fully susceptible populations based on area-specific populations and age structures estimated for 1991 [30]. Each population was stratified into 320 age classes (monthly strata sup to age 20 years, and yearly strata until age 100 years) using smoothing splines. In order to move beyond the transient non- seasonal outbreaks to populations representing endemic rubella, we then seed infected individuals into each population and iteratively simulated rubella dynamics for four 20 year increments rescaling the mean WAIFW (*β*¯*a,j* ) by the assumed *R*0 and the population by the 1991 population and age structure at the end of each 20 year cycle. The result was populations representing endemic rubella in 1991 for each states’ rural and urban area (i.e., **n**(1)).

We then simulated the population forward 56 years (1344 time-steps), including rescaling the total population after 20 years (480 time-steps) based on the 2011 census [31], while maintaining area-specific age structures (Supplemental Figure S3).

Population survival rates per age class (i.e., *sa*) were estimated by matching each area’s 2010 life expectancy [32] to the United Nations ‘Coale-Demeny East’ life table model [33], and holding these rates constant over time. Life expectancy for rural and urban Uttarakhand and the north eastern states with the exception of Assam (i.e., Arunachal Pradesh, Manipur, Meghalaya, Mizoram, Nagaland, Tripura) assumed the life expectancy of rural and urban India, respectively.

The number of births per time-step *t* (i.e., *bt*) were estimated by multi- plying the estimated birth rate per time-step *t* by the total population at time-step *t* (**n**(*t*)). Crude birth rates (CBR, i.e., yearly rate of births per 1000) between 1991 and 2013 were extracted for all 52 areas from the Sample Registration System [34, 35]. The CBR for Chhattisgarh, Jharkhand, and Uttarakhand prior to 1999 were input from Madhya Pradesh, Bihar, and Uttar Pradesh CBR, respectively. India is in phase II of the fertility transition, which is falling fertility [36, 37]. We estimated past, current, and future birth rates by fitting a negative sigmoid function to the CBR data over time. If by 2013, the area’s births had not yet dropped below the replacement level total fertility rate (TFR) of 2.1, we estimated the lower asymptote as the minimum CBR equal to a replacement level TFR of 2.1 times the average number of women per age year between 15 and 40 years old, divided by the population total per the 2011 census [31]. If the area’s TFR fell below a replacement level of 2.1 as of 2013, then the lower asymptote CBR was set to the recent CBR minus one. See Supplemental Figure S4a,b for the estimated 2015 crude birth rates for each area.

Estimation of CRS required area-specific age-specific fertility rate (ASFR). ASFRs were extracted from the 2012 Sample Registration System [38] and held constant over time. Due to missing data, the following small states took on India’s average rural ASFR and urban ASFR for their rural and urban counterparts: Uttarakhand and the north eastern states with exception of Assam (i.e., Arunachal Pradesh, Manipur, Meghalaya, Mizoram, Nagaland, Tripura).

# Supplementary Results

Table S1: State level covariates associated with Figure 1 in the main text. Column 2

1. Rural private-sector routine RCV coverage (as a proportion). Column 3 (b) Urban private-sector routine RCV coverage (as a proportion). Column 4 (c) Rural public-sector routine MR coverage (as a proportion). Column 5 (d) Urban public-sector routine MR coverage (as a proportion). All coverage estimates were extracted from the Rapid Survey on Children 2013-14 [39]. To estimate private-sector routine RCV coverage we assumed that any child who received their vaccinations in a private healthcare center received RCV. To estimate public-sector routine MR coverage we assumed that current MCV1 coverage estimates reflect future routine MR coverage estimates.

| State | (a) Rural private-sector RCV coverage | (b) Urban private-sector RCV coverage | (c) Rural public-sector  MR coverage | (d) Urban public-sector MR coverage |
| --- | --- | --- | --- | --- |
| Andhra Pradesh | 0.138 | 0.29 | 0.883 | 0.825 |
| Arunachal Pradesh | 0.019 | 0 | 0.842 | 0.907 |
| Assam | 0.025 | 0.155 | 0.786 | 0.792 |
| Bihar | 0.01 | 0.107 | 0.689 | 0.67 |
| Chhattisgarh | 0.017 | 0.121 | 0.847 | 0.893 |
| Gujarat | 0.082 | 0.299 | 0.766 | 0.863 |
| Haryana | 0.038 | 0.33 | 0.813 | 0.777 |
| Himachal Pradesh | 0.044 | 0.105 | 0.937 | 0.867 |
| Jammu & Kashmir | 0.057 | 0.112 | 0.731 | 0.834 |
| Jharkhand | 0.078 | 0.274 | 0.738 | 0.824 |
| Karnataka | 0.07 | 0.296 | 0.89 | 0.85 |
| Kerala | 0.411 | 0.632 | 0.936 | 0.932 |
| Madhya Pradesh | 0.008 | 0.103 | 0.738 | 0.776 |
| Maharashtra | 0.085 | 0.278 | 0.951 | 0.9 |
| Manipur | 0.025 | 0.092 | 0.723 | 0.789 |
| Meghalaya | 0.039 | 0.228 | 0.69 | 0.648 |
| Mizoram | 0 | 0.141 | 0.949 | 0.952 |
| Nagaland | 0.037 | 0.2 | 0.52 | 0.549 |
| Odisha | 0.007 | 0.067 | 0.755 | 0.755 |
| Punjab | 0.106 | 0.361 | 0.888 | 0.836 |
| Rajasthan | 0.032 | 0.133 | 0.596 | 0.765 |
| Tamil Nadu | 0.134 | 0.304 | 0.921 | 0.911 |
| Tripura | 0 | 0.069 | 0.735 | 0.823 |
| Uttar Pradesh | 0.036 | 0.199 | 0.601 | 0.694 |
| Uttarakhand | 0.066 | 0.195 | 0.715 | 0.812 |
| West Bengal | 0.004 | 0.089 | 0.857 | 0.862 |

Table S2: Results of simulated rubella dynamics assuming an R0 of 5 and private-sector vaccination since 1993 associated with Figure 2 in the main text. Column 2 (a) Rural estimated 2016 number of CRS cases by state determined by ‘private-sector vaccine’ scenario. Column 3 (b) Urban estimated 2016 number of CRS cases by state determined by ‘private-sector vaccine’ scenario. Column 4 (c) Rural estimated 2016 CRS incidence per 100,000 live births by state determined by ‘private-sector vaccine’ scenario. Column 5 (d) Urban estimated 2016 CRS incidence per 100,000 live births by state determined by ‘private-sector vaccine’ scenario.

| State | (a) Rural # of CRS cases | (b) Urban # of CRS cases | (c) Rural CRS incidence  per 100,000 live births | (d) Urban CRS incidence per 100,000 live births |
| --- | --- | --- | --- | --- |
| Andhra Pradesh | 971 | 425 | 98 | 87 |
| Arunachal Pradesh | 12 | 3 | 56 | 73 |
| Assam | 436 | 56 | 65 | 78 |
| Bihar | 1310 | 150 | 48 | 60 |
| Chhattisgarh | 271 | 81 | 52 | 74 |
| Gujarat | 459 | 353 | 60 | 70 |
| Haryana | 203 | 113 | 54 | 64 |
| Himachal Pradesh | 72 | 4 | 62 | 57 |
| Jammu & Kashmir | 88 | 26 | 57 | 58 |
| Jharkhand | 346 | 113 | 54 | 79 |
| Karnataka | 566 | 340 | 73 | 81 |
| Kerala | 202 | 46 | 77 | 20 |
| Madhya Pradesh | 737 | 258 | 48 | 65 |
| Maharashtra | 874 | 636 | 79 | 76 |
| Manipur | 22 | 9 | 76 | 74 |
| Meghalaya | 30 | 6 | 50 | 77 |
| Mizoram | 8 | 6 | 72 | 82 |
| Nagaland | 17 | 7 | 80 | 83 |
| Odisha | 439 | 77 | 63 | 73 |
| Punjab | 197 | 96 | 63 | 61 |
| Rajasthan | 722 | 239 | 48 | 61 |
| Tamil Nadu | 490 | 437 | 83 | 80 |
| Tripura | 31 | 8 | 78 | 71 |
| Uttar Pradesh | 2139 | 605 | 47 | 56 |
| Uttarakhand | 91 | 44 | 70 | 93 |
| West Bengal | 1104 | 304 | 97 | 94 |

Table S3: The number of months until rubella outbreak (5 rubella cases per 100,000 population) post-vaccine introduction, by *R*0 and for each state by rural and urban area. The table also displays the proportion of rubella cases required to be reported in order to pick up a rubella outbreak of greater than or equal to 5 per 100,000 population. The results are determined by the ‘60% catch-up + routine vaccine’ scenario. Note: our definition of reporting rate is simply five divided by the total number of cases in the outbreak and does not reflect typical surveillance terminology which defines reporting rate as the proportion of reporting sites that report cases.

| State Rural and Urban Areas | # months  R0=5 | until rubella  R0=7 | outbreak (reporting rate)  R0=9 R0=11 | |
| --- | --- | --- | --- | --- |
| Andhra Pradesh Rural | - | - | - | 106 (0.25) |
| Andhra Pradesh Urban | - | - | 146 (0.66) | 106 (0.09) |
| Arunachal Pradesh Rural | - | - | 93 (0.1) | 78 (0.02) |
| Arunachal Pradesh Urban | - | - | - | NA (0.84) |
| Assam Rural | - | 107 (0.28) | 81 (0.06) | 69 (0.03) |
| Assam Urban | - | - | 104 (0.06) | 82 (0.05) |
| Bihar Rural | 108 (0.24) | 78 (0.01) | 64 (0) | 54 (0) |
| Bihar Urban | 144 (0.32) | 91 (0.03) | 75 (0.03) | 64 (0) |
| Chhattisgarh Rural | - | 119 (1) | 90 (0.13) | 76 (0.04) |
| Chhattisgarh Urban | - | - | - | 106 (0.51) |
| Gujarat Rural | - | 106 (0.15) | 80 (0.04) | 68 (0.02) |
| Gujarat Urban | - | - | - | 107 (0.19) |
| Haryana Rural | - | 120 (0.48) | 90 (0.04) | 76 (0.01) |
| Haryana Urban | - | 158 (0.35) | 105 (0.04) | 83 (0.03) |
| Himachal Pradesh Rural | - | - | - | 119 (0.57) |
| Himachal Pradesh Urban | - | - | - | 120 (0.41) |
| Jammu & Kashmir Rural | - | 133 (0.36) | 94 (0.06) | 79 (0.01) |
| Jammu & Kashmir Urban | - | - | 133 (0.46) | 103 (0.16) |
| Jharkhand Rural | - | 91 (0.05) | 76 (0.02) | 64 (0.02) |
| Jharkhand Urban | - | - | 132 (0.41) | 95 (0.07) |
| Karnataka Rural | - | - | 120 (0.55) | 92 (0.12) |
| Karnataka Urban | - | - | - | 117 (0.26) |
| Kerala Rural | - | - | - | - |
| Kerala Urban | - | - | - | - |
| Madhya Pradesh Rural | 132 (0.5) | 82 (0.09) | 68 (0.02) | 61 (0.02) |
| Madhya Pradesh Urban | - | 131 (0.29) | 92 (0.05) | 78 (0.01) |
| Maharashtra Rural | - | - | - | - |
| Maharashtra Urban | - | - | - | - |
| Manipur Rural | - | 121 (0.29) | 92 (0.03) | 78 (0.01) |
| Manipur Urban | - | 146 (0.67) | 103 (0.13) | 80 (0.03) |
| Meghalaya Rural | 121 (0.29) | 81 (0.03) | 67 (0.01) | 57 (0.01) |
| Meghalaya Urban | 210 (0.61) | 117 (0.04) | 91 (0.01) | 77 (0) |
| Mizoram Rural | - | - | - | 108 (0.43) |
| Mizoram Urban | - | - | - | - |
| Nagaland Rural | 120 (0.1) | 89 (0.04) | 69 (0.01) | 63 (0.01) |
| Nagaland Urban | 146 (0.08) | 103 (0.04) | 80 (0) | 68 (0) |
| Odisha Rural | - | 105 (0.1) | 80 (0.03) | 68 (0.02) |
| Odisha Urban | - | 145 (0.42) | 96 (0.11) | 80 (0.02) |
| Punjab Rural | - | - | - | 104 (0.13) |
| Punjab Urban | - | - | - | 130 (0.32) |
| Rajasthan Rural | 94 (0.05) | 69 (0.01) | 57 (0) | 51 (0) |
| Rajasthan Urban | - | 107 (0.14) | 81 (0.03) | 68 (0.01) |
| Tamil Nadu Rural | - | - | - | - |
| Tamil Nadu Urban | - | - | - | - |
| Tripura Rural | - | 130 (0.25) | 93 (0.05) | 78 (0.01) |
| Tripura Urban | - | - | 132 (0.27) | 103 (0.11) |
| Uttar Pradesh Rural | 104 (0.03) | 76 (0.03) | 63 (0.01) | 53 (0) |
| Uttar Pradesh Urban | 160 (0.44) | 94 (0.06) | 78 (0.01) | 65 (0) |
| Uttarakhand Rural | - | 106 (0.46) | 81 (0.17) | 69 (0.1) |
| Uttarakhand Urban | - | - | 130 (0.2) | 94 (0.23) |
| West Bengal Rural | - | - | 120 (0.5) | 93 (0.12) |
| West Bengal Urban | - | - | - | 145 (0.88) |
| Average | 127 (0.27) | 107 (0.15) | 91 (0.045) | 79 (0.03) |

# References

- 1. **Metcalf C**, *et al.* Structured models of infectious disease: Inference with discrete data. *Theoretical Population Biology* 2012; **82**: 275–282.
  2. **Klepac P**, *et al.* Stage-structured transmission of phocine distemper virus in the dutch 2002 outbreak. *Proceedings of the Royal Society of London. Series B (Biological Sciences)* 2009; **276**: 2469–2476.
  3. **Klepac P**, **Caswell H**. The stage-structured epidemic: linking disease and demography with a multi-state matrix approach model. *Theoretical Ecology* 2011; **4**: 301–319.
  4. **Metcalf C**, *et al.* Impact of birth rate, seasonality and transmission rate on minimum levels of coverage needed for rubella vaccination. *Epi- demiology and Infection* 2012; **140**: 2290–2301.
  5. **Bjornstad O**, **Finkenstadt B**, **Grenfell B**. Dynamics of measles epidemics: Estimating scaling of transmission rates using a time series sir model. *Ecological Monographs* 2002; **72**: 169–184.
  6. **Finkenstadt B**, **Grenfell B**. Time series modelling of childhood dis- eases: a dynamical systems approach. *Journal of the Royal Statistical Society Series C-Applied Statistics* 2000; **49**: 187–205.
  7. **Glass K**, **Xia Y**, **Grenfell B**. Interpreting time-series analyses for continuous-time biological models-measles as a case study. *Journal of Theoretical Biology* 2003; **223**: 19–25.
  8. **Metcalf C**, *et al.* Rubella metapopulation dynamics and importance of spatial coupling to the risk of congenital rubella syndrome in Peru. *Journal of the Royal Society Interface* 2011; **8**: 369–376.
  9. **Waaijenborg S**, *et al.* Waning of maternal antibodies against measles, mumps, rubella, and varicella in communities with contrasting vaccina- tion coverage. *Journal of Infectious Diseases* 2013; **208**: 10–16.
  10. **Nicoara C**, *et al.* Decay of passively acquired maternal antibodies against measles, mumps, and rubella viruses. *Clinical and Diagnostic Laboratory Immunology* 1999; **6**: 868–871.
  11. **Vynnycky E**, *et al.* Using seroprevalence and immunisation coverage data to estimate the global burden of congenital rubella syndrome, 1996- 2010: A systematic review. *PLoS One* 2016; **11**: e0149160.
  12. **Seth P**, **Balaya S**, **Mohapatra LN**. Sero-epidemiological study of rubella infection in female subjects of Delhi and its surrounding villages. *Indian Journal of Medical Research* 1971; **59**: 190–4.
  13. **Mathur A**, **Chaturve U**, **Mehrotra RM**. Serological study for preva- lence of rubella at Lucknow. *Indian Journal of Medical Research* 1974; **62**: 307–312.
  14. **Pal SR**, *et al.* Serological investigation of rubella-virus infection in and around Chandigarh - preliminary communication. *Indian Journal of Medical Research* 1974; **62**: 240–245.
  15. **Chakravarty MS**, *et al.* Seroepidemiological study of rubella in Cal- cutta. *Indian Journal of Medical Research* 1976; **64**: 87–92.
  16. **Khare S**, *et al.* Lowered immunity status of rubella virus infection in pregnant women. *Journal of Communicable Diseases* 1987; **19**: 391–5.
  17. **Anderson R**, **May R**. *Infectious Diseases of Humans: Dynamics and Control*. New York: Oxford University Press, 1991.
  18. **The World Bank Group**. Data: Life expectancy at birth, total (years), 2015. URL [http://data.worldbank.org/indicator/SP.DYN.](http://data.worldbank.org/indicator/SP.DYN) LE00.IN. Accessed: August 30, 2016.
  19. **Edmunds W**, *et al.* The pre-vaccination epidemiology of measles, mumps and rubella in Europe: implications for modeling studies. *Epi- demiology and Infection* 2000; **125**: 635–650.
  20. **Cutts F**, *et al.* Sero-epidemiology of rubella in the urban population of Addis Ababa, Ethiopia. *Epidemiology and Infection* 2000; **124**: 467– 479.
  21. **Lessler J**, **Metcalf C**. Balancing evidence and uncertainty when con- sidering rubella vaccine introduction. *PLoS One* 2013; **8**: e67639.
  22. **Keeling M**, **Rohani P**, **Grenfell B**. Seasonally forced disease dy- namics explored as switching between attractors. *Physica D* 2001; **148**: 317–335.
  23. **Metcalf C**, *et al.* The epidemiology of rubella in Mexico: seasonality, stochasticity and regional variation. *Epidemiology and Infection* 7 2011; **139**: 1029–1038.
  24. **Wesolowski A**, *et al.* Introduction of rubella-containing-vaccine to Madagascar: implications for roll-out and local elimination. *Journal of the Royal Society Interface* 2016; **13**.
  25. **Mossong J**, *et al.* Social contacts and mixing patterns relevant to the spread of infectious diseases. *PLoS Medicine* 2008; **5**: 381–391.
  26. **Horby P**, *et al.* Social contact patterns in Vietnam and implications for the control of infectious diseases. *PLoS One* 2011; **6**: e16965.
  27. **DeStefano F**, *et al.* Factors associated with social contacts in four communities during the 2007-2008 influenza season. *Epidemiology and Infection* 2011; **139**: 1181–90.
  28. **Lessler J**, *et al.* Maintaining high rates of measles immunization in Africa. *Epidemiology and Infection* 2011; **139**: 1039–1049.
  29. **Boulianne N**, *et al.* Measles, mumps, and rubella antibodies in chil- dren 5-6 years after immunization: effect of vaccine type and age at vaccination. *Vaccine* 1995; **13**: 1611–6.
  30. **Office of the Registrar General & Census Commissioner**. C-5: Single year age returns tables (india/states), 1991. URL [http://www.](http://www/) censusindia.gov.in/DigitalLibrary/TableSeries.aspx. Accessed: March, 1 2015.
  31. **Office of the Registrar General & Census Commissioner**. Population enumeration data: Single year age data - C13 table (India/States/UT), 2011. URL <http://www.censusindia.gov.in/> 2011census/population\_enumeration.aspx. Accessed: June 5, 2014.
  32. **Office of the Registrar General & Census Commis- sioner**. SRS based abridged life tables, 2003-2007 to 2006-2010, 2012. URL [http://www.censusindia.gov.in/vital\_statistics/](http://www.censusindia.gov.in/vital\_statistics/) SRS\_Based/SRS\_Based.html. Accessed: June 6, 2014.
  33. **UN Population Division**. World population prospects 2012: Extended model life tables, 2010. URL <http://esa.un.org/> wpp/Model-Life-Tables/data/MLT\_UN2010-130\_1y.xls. Accessed: June 5, 2014.
  34. **Office of Registrar General & Census Commissioner**. *Vital rates of India, 1971 to 1996, based on the sample registration system (SRS)*. New Delhi, India: Registrar General, 1998.
  35. **Office of Registrar General & Census Commissioner**. Sample Registration System (SRS) Bulletins, 1999-2014. URL [http://www.censusindia.gov.in/vital\_statistics/SRS\](http://www.censusindia.gov.in/vital\_statistics/SRS\)

_Bulletins/Bulletins.html.

- 1. **United Nations**. World population prospects: The 2015 revision, DVD edition, 2015.
  2. **United Nations and Department of Economic and Social Af- fairs and Population Division**. World population prospects. Proba- bilistic projections, 2015.
  3. **Office of Registrar General & Census Commissioner**. SRS statistical report: SRS statistical report tables, 2012. URL [http://www.censusindia.gov.in/vital\_statistics/SRS\](http://www.censusindia.gov.in/vital\_statistics/SRS\)

_Reports\_2012.html. Accessed: January 2, 2015.

- 1. **Ministry of Women and Child Development**, **Govern- ment of India**. Rapid Survey on Children (2013-14): In- dia and state fact sheets, 2016. URL <http://wcd.nic.in/acts/> rapid-survey-children-rsoc-2013-14. Accessed: January 4, 2017.
