# Supplementary figures and images for: Rubella vaccination in India: identifying broad consequences of vaccine introduction and key knowledge gaps
Source: Epidemiol Infect. 2017 Dec 4;146(1):65–77. doi: 10.1017/S0950268817002527 (PMC6024169; doi:10.1017/S0950268817002527)

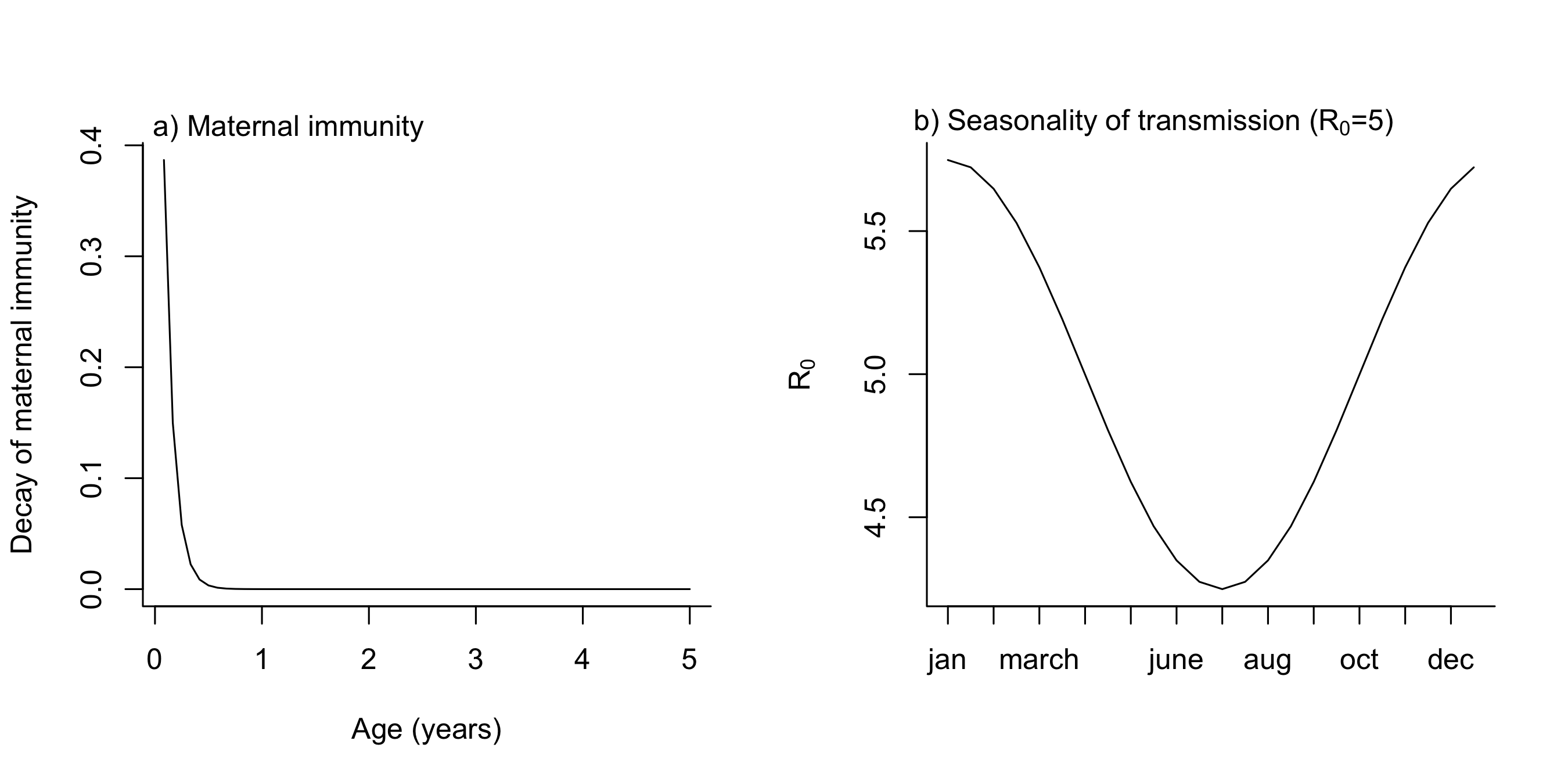

Supplement: Supplementary file 1 [file S0950268817002527sup001.zip › S0950268817002527sup001/SFig1.tif]

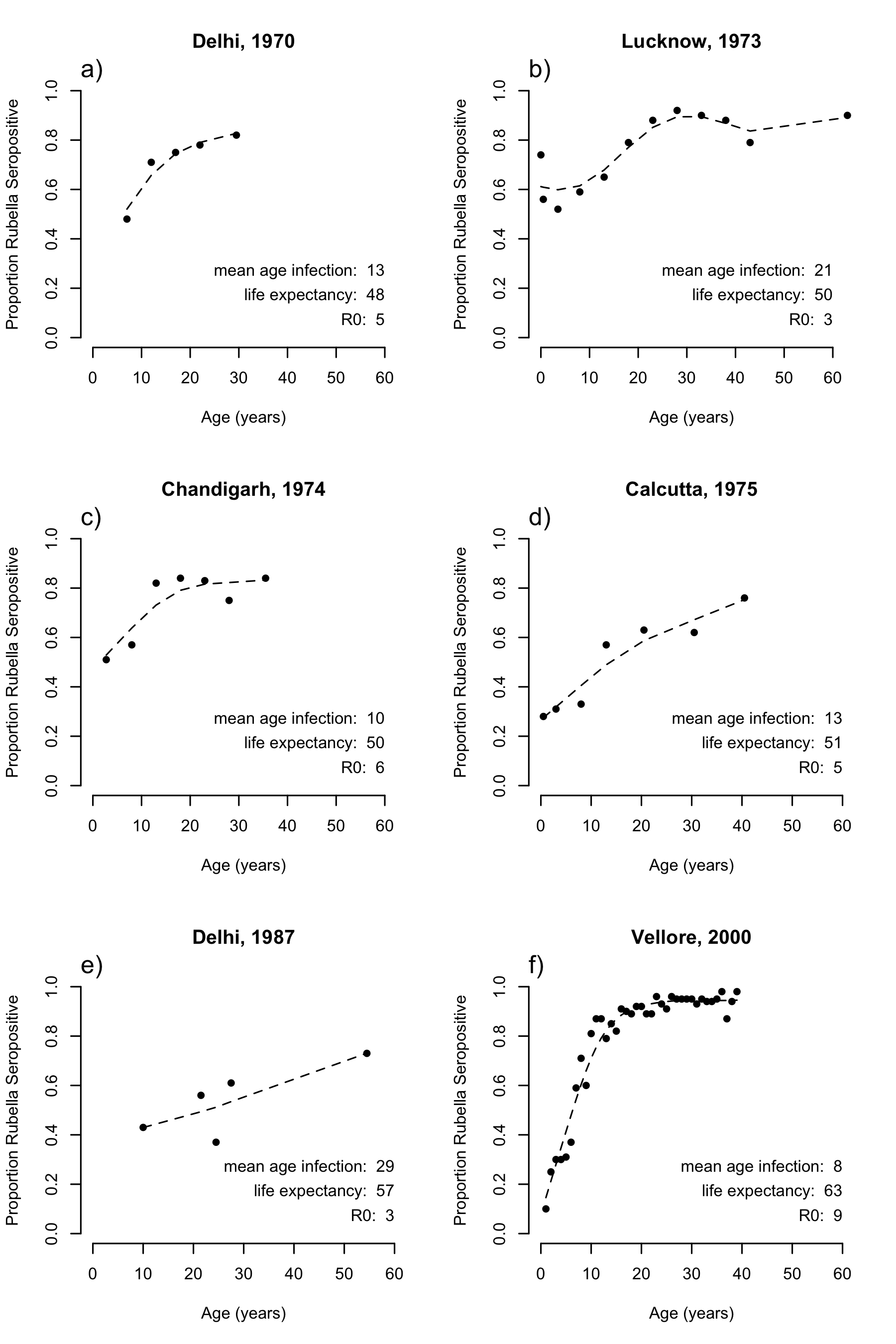

Supplement: Supplementary file 1 [file S0950268817002527sup001.zip › S0950268817002527sup001/SFig2.tif]

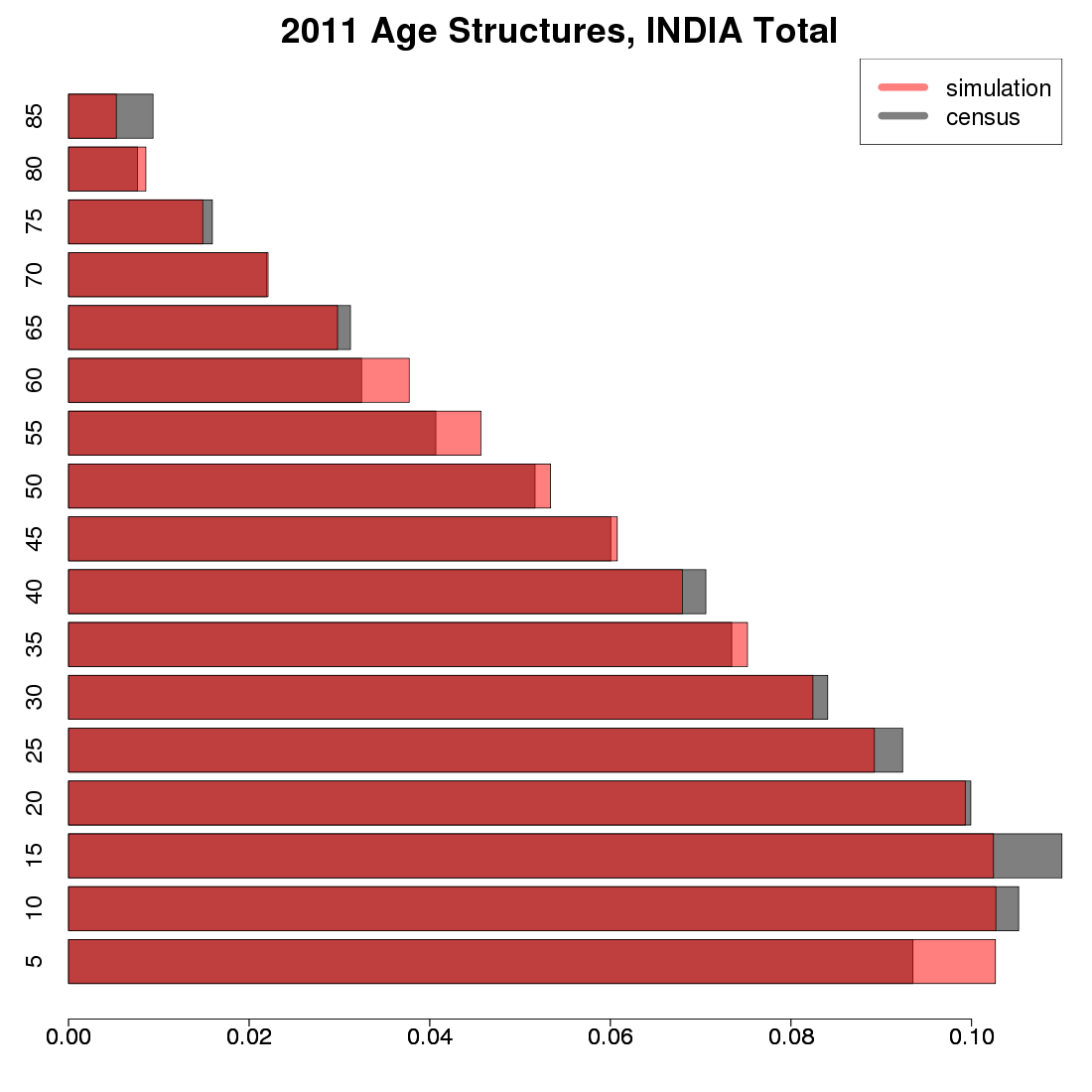

Supplement: Supplementary file 1 [file S0950268817002527sup001.zip › S0950268817002527sup001/SFig3.tif]

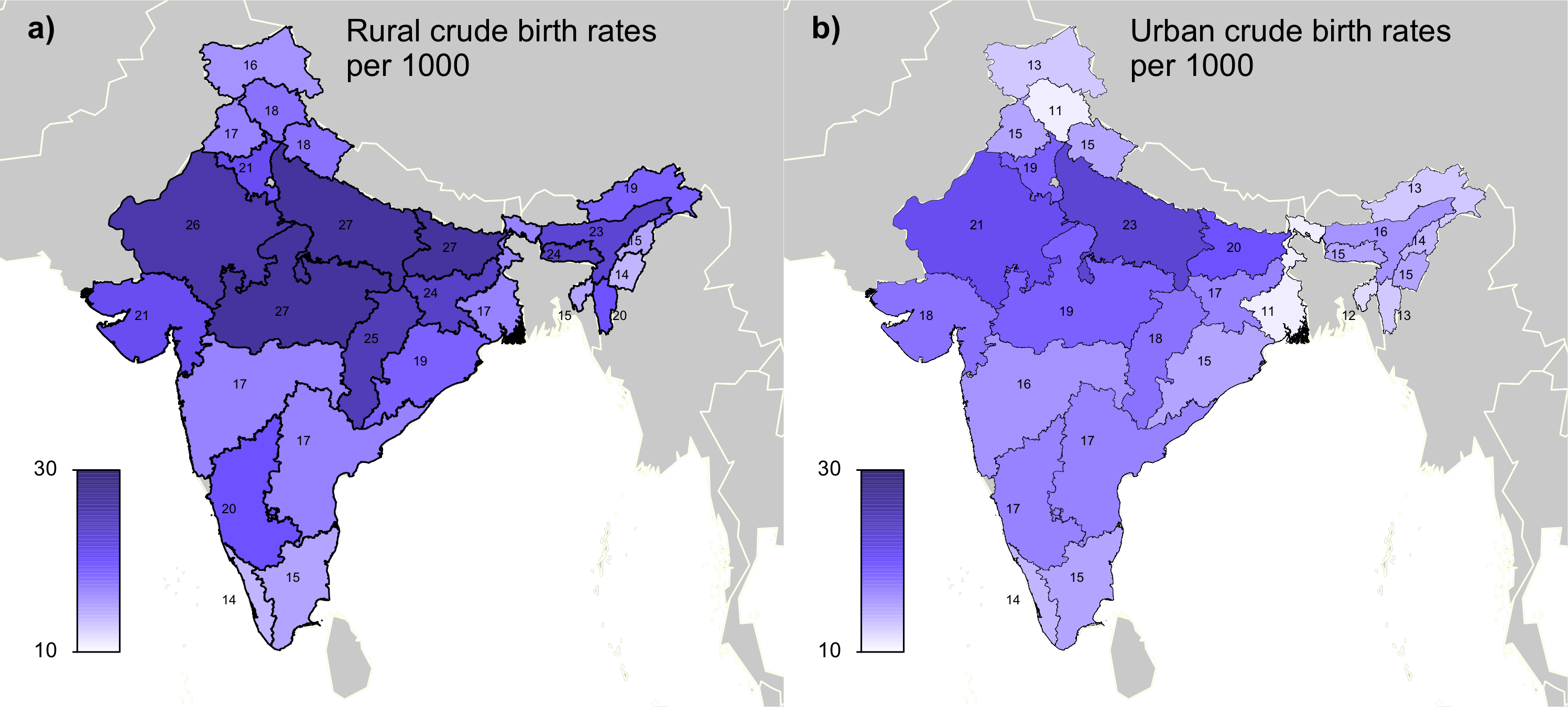

Supplement: Supplementary file 1 [file S0950268817002527sup001.zip › S0950268817002527sup001/SFig4.tif]

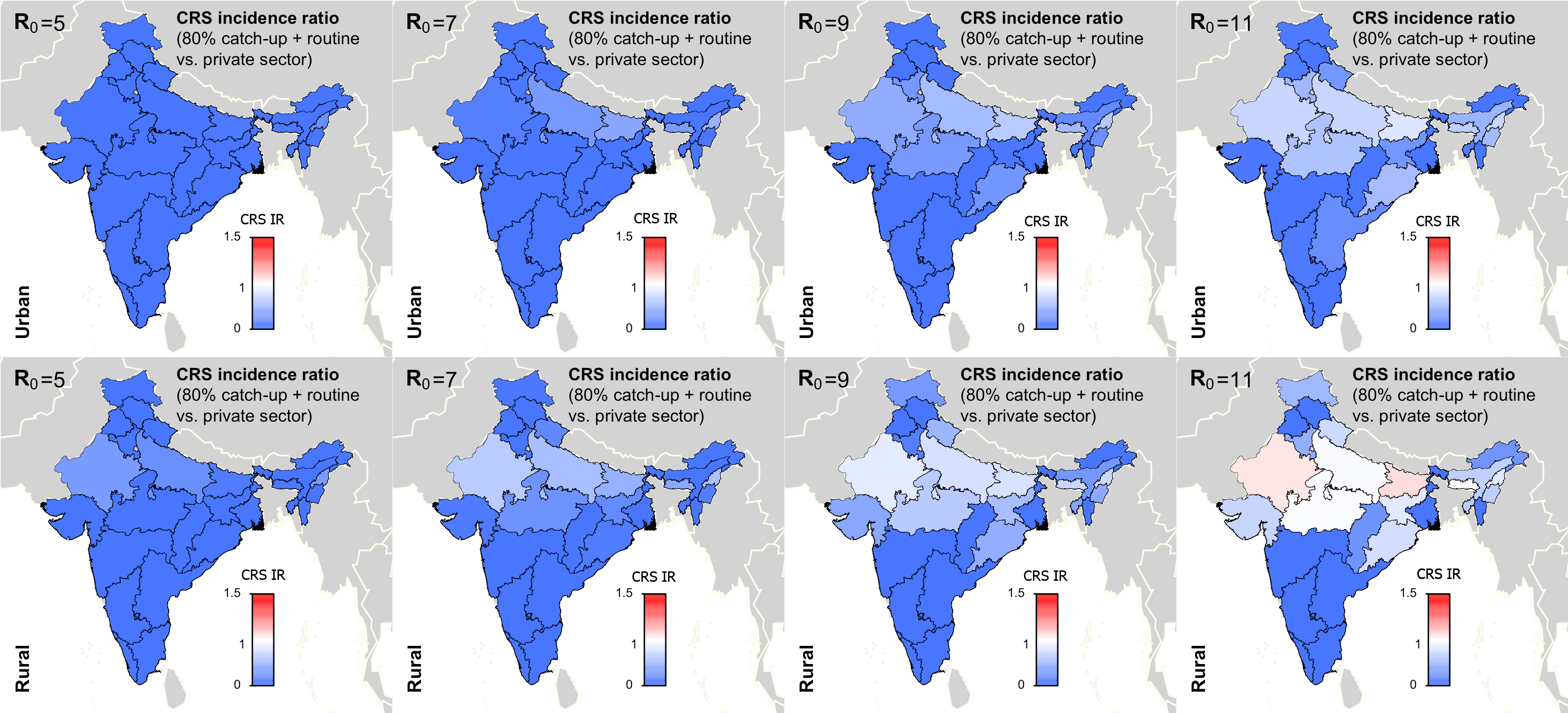

Supplement: Supplementary file 1 [file S0950268817002527sup001.zip › S0950268817002527sup001/SFig7.tif]

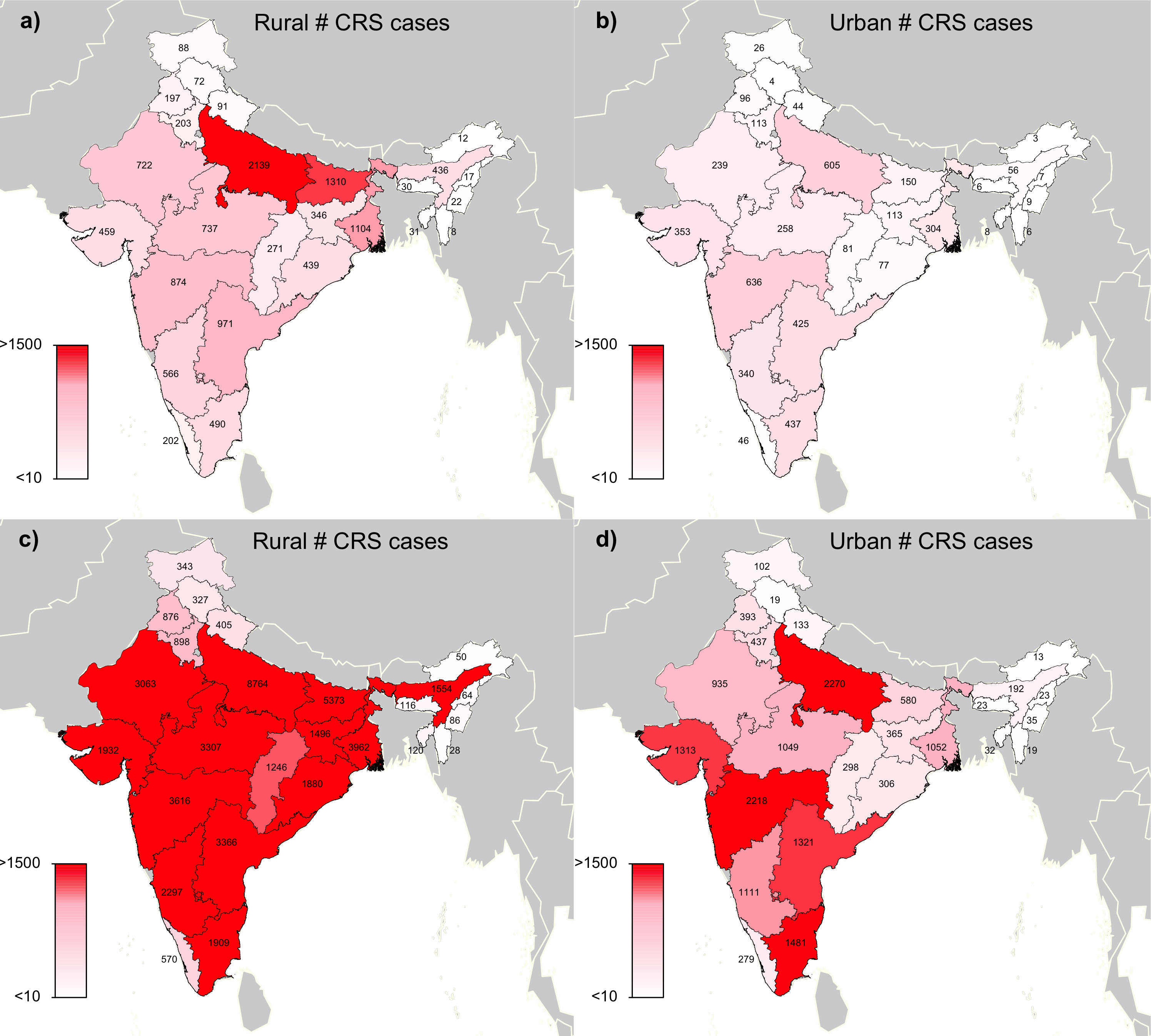

Supplement: Supplementary file 1 [file S0950268817002527sup001.zip › S0950268817002527sup001/SFig8.tif]

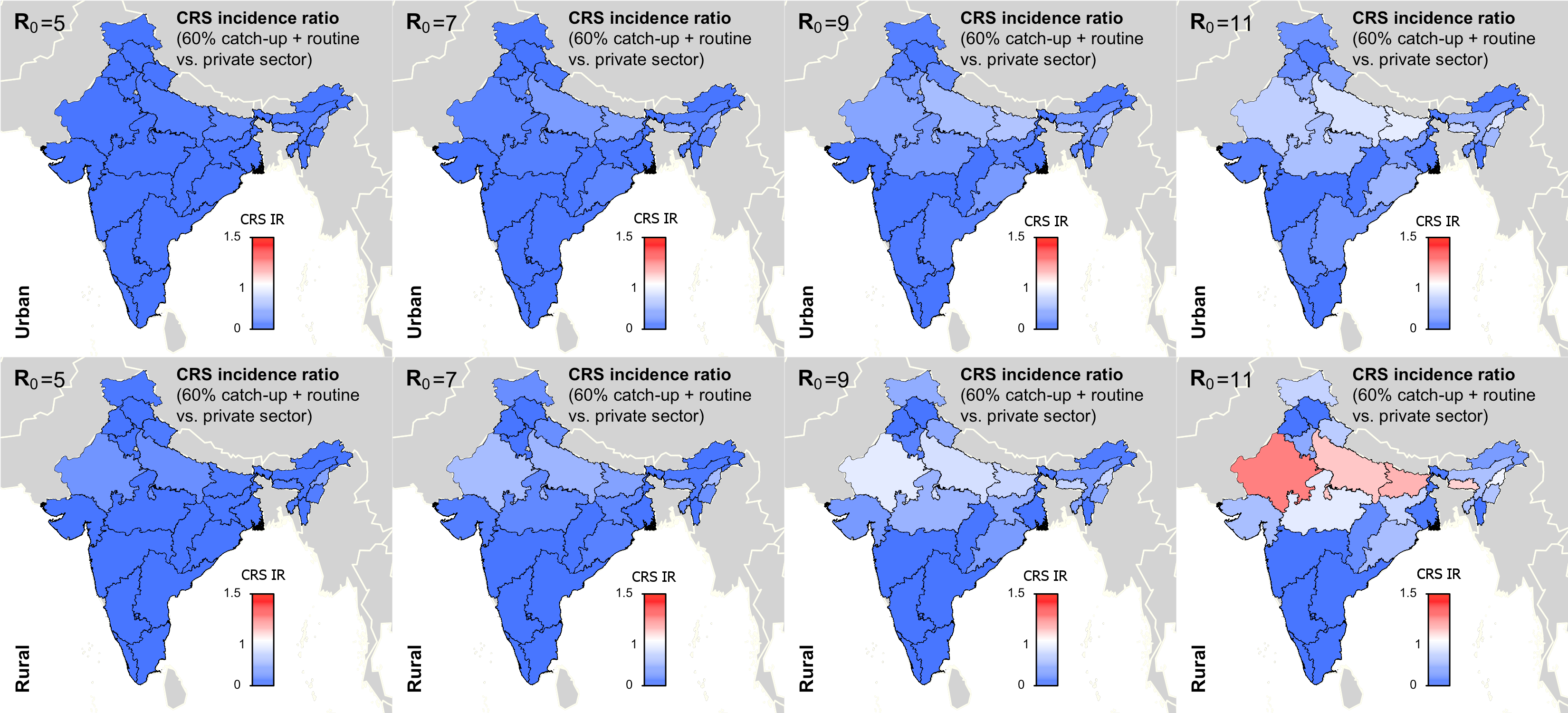

Supplement: Supplementary file 1 [file S0950268817002527sup001.zip › S0950268817002527sup001/SFig9.tif]
